# A scoring model based on bacterial lipopolysaccharide-related genes to predict prognosis in NSCLC

**DOI:** 10.3389/fgene.2024.1408000

**Published:** 2024-11-14

**Authors:** Nandi Bao, Xinxin Zhang, Chenyu Lin, Feng Qiu, Guoxin Mo

**Affiliations:** ^1^ Senior Department of Cardiology, The Sixth Medical Center of PLA General Hospital, Beijing, China; ^2^ Department of Pulmonary and Critical Care Medicine, The Eighth Medical Center of the PLA General Hospital, Beijing, China; ^3^ Senior Department of Neurology, First Medical Center of the PLA General Hospital, Beijing, China

**Keywords:** non-small cell lung cancer, lipopolysaccharide-related genes, prognosis, therapeutic response, tumor immune environment, risk score

## Abstract

**Background:**

Non-small cell lung cancer (NSCLC) has high incidence and mortality rates. The discovery of an effective biomarker for predicting prognosis and treatment response in patients with NSCLC is of great significance. Bacterial lipopolysaccharide-related genes (LRGs) play a critical role in tumor development and the formation of an immunosuppressive microenvironment; however, their relevance in NSCLC prognosis and immune features is yet to be discovered.

**Methods:**

Differentially expressed LRGs associated with NSCLC prognosis were identified in the TCGA dataset. Prognostic LRG scoring and nomogram models were established using single-variable Cox regression, Least Absolute Shrinkage, and Selection Operator (LASSO) regression. The prognostic value of the scoring and nomogram models was evaluated using Kaplan-Meier (KM) analysis and further validated using an external dataset. Patients were stratified into high- and low-risk groups based on the nomogram score, and drug sensitivity analysis was performed. Additionally, clinical characteristics, mutation features, immune infiltration characteristics, and responses to immunotherapy were compared between the two groups.

**Results:**

We identified 15 differentially expressed LRGs associated with NSCLC prognosis. A prognostic prediction model consisting of 6 genes (VIPR1, NEK2, HMGA1, FERMT1, SLC7A, and TNS4) was established. Higher LRG scores were associated with worse clinical prognosis and were independent prognostic factors for NSCLC. Subsequently, a clinical risk prediction nomogram model for NSCLC was constructed, incorporating the status of patients with tumor burden, tumor T-stage, and LRG scores. The nomogram model demonstrated good predictive performance upon validation. Additionally, NSCLC patients classified as high risk based on the model’s predictions exhibited not only a poorer prognosis but also a more pronounced inflammatory immune microenvironment phenotype than low-risk patients. Furthermore, high-risk patients showed disparate predicted responses to various drugs and immunotherapies compared with low-risk patients.

**Conclusion:**

The LRGs scoring model can serve as a biomarker that contributes to the establishment of a reliable prognostic risk-prediction model, potentially facilitating the development of personalized treatment strategies for patients with NSCLC.

## Background

Lung cancer, or bronchogenic carcinoma, is the leading cause of oncological mortality globally and originates from the bronchi or pulmonary parenchyma. Based on the pathological morphology and malignancy, it can be classified into two primary subtypes: small cell lung cancer (SCLC) and non-small cell lung cancer (NSCLC). NSCLC represents the majority of lung cancers, constituting approximately 80%–85% of all cases (Michelotti et al., 2022). Systemic therapies, including chemotherapy, targeted therapy, immunotherapy, and anti-angiogenic drugs, are the principal treatment modalities for most patients with advanced NSCLC (Roller et al., 2022). Despite these measures, the prognosis of patients with advanced NSCLC remains unfavorable (Otano et al., 2023).

The tumor microenvironment (TME), an entity increasingly acknowledged as a pivotal determinant of oncogenesis, progression, and therapeutic response in cancer, is composed of intratumoral microbes and their metabolites. Current research has elucidated the presence of unique microbial milieu features across a spectrum of diverse tumor types, revealing that each tumor’s microbial signature is distinct (Ma et al., 2021). Given that the microbial composition within tumors serves as a potential microbiome-based oncology diagnostic tool, its efficacy as a cancer prognostic biomarker is promising (Poore et al., 2020; Xue et al., 2023). Additionally, discernible differences in microbiome profiles between tumors and normal tissues highlight their potential as novel therapeutic targets for the treatment of various malignancies, particularly lung cancer (Lee et al., 2016; Greathouse et al., 2018). The lungs have an immune defense system chiefly orchestrated by macrophages, whereas the pulmonary epithelium exhibits an inherent capacity to eliminate microbial intruders. Therefore, although the lung mucosal surface is in direct contact with the external environment and exposed to exogenous microbes, the microbial burden detected in healthy lungs remains minimal. Owing to the extensive interconnectivity and bidirectional interactions between the gastrointestinal tract and the respiratory system, the gut-lung axis (GLA) is implicated in the pathophysiology of an array of gut and lung disorders. The gut microbiota can modulate the pathological processes of distal neoplasms, particularly lung cancer, through various pathways. While esophageal contents may enter the pulmonary system via aspiration, the intricate interconnections between the intestines and lungs through the lymphatic and circulatory systems suggest that stimuli affecting the local immune system can propagate systemic effects (Gill et al., 2010). These investigations allowed us to postulate that the unique microbiome composition and immune environment within lung cancer tissues, including NSCLC, may play a role in tumor development and progression through various mechanisms.

Recent research has revealed a notable increase in the prevalence of the *Phylum Proteobacteria*, which includes *Escherichia coli*, in lung cancer tissues compared to healthy controls (Greathouse et al., 2018). Additionally, investigations have demonstrated an augmented representation of the Veillonellaceae family in lung tumor samples (Peters et al., 2022). The Veillonellaceae family and Proteobacteria phyla were classified under the gram-negative bacterial category. Lipopolysaccharide (LPS), a pivotal constituent of the outer membrane of gram-negative bacteria, is the primary virulence determinant. LPS has been implicated in the pathogenesis of many diseases through the induction of immune responses, alteration of cellular metabolism, and regulation of cytokine expression profiles (Chiariotti et al., 2016). Furthermore, they can interfere with the onset and progression of diseases by modulating host gene expression patterns (Yi et al., 2016). Genes whose expression levels are modulated by LPS stimulation are designated as lipopolysaccharide (LPS)-related genes (LRGs). Studies have demonstrated that LPS promotes the secretion of pro-inflammatory cytokines by oncogenic cells, eliciting localized inflammatory responses and promoting the advancement of tumorigenic processes (Xu et al., 2019). LPS can facilitate tumor cell adhesion by upregulating the oncogenic gene SPP1, thus promoting metastasis of malignant tumors, especially NSCLC (Tang et al., 2021). Consequently, it is reasonable to speculate that LPS and its downstream genetic effects influence the prognosis and treatment outcomes for NSCLC patients with NSCLC.

In this study, LRGs were used to explore putative molecular underpinnings of the NSCLC microbiome. Furthermore, a novel predictive model for the prognostic risk of NSCLC was constructed and used to predict LRGs that correlated with prognostic outcomes.

## Methods

### Data acquisition and filtering

RNA sequencing gene expression data (FPKM) and clinical information of patients with NSCLC were downloaded from The Cancer Genome Atlas (TCGA) database (https://portal.gdc.cancer.gov/). After standardization and selection of samples with available prognostic information, a cohort of 1,109 samples, including 1,001 NSCLC cases and 108 normal controls, was used as the training set. The clinical data for this training set are provided in Supplementary Table S1. The GSE37745 cohort was downloaded from the NCBI Gene Expression Omnibus (GEO) database (https://www.ncbi.nlm.nih.gov/geo/), which contained 196 samples, each of which contained associated survival information. Preprocessed, normalized, and log2-transformed expression matrices were obtained for the probes. Additionally, we downloaded platform annotation files to convert the probe expression matrix into gene symbols. This dataset was used as the validation set. The detailed clinical information of the patients is displayed in Supplementary Table S2. First, we conducted a differential expression analysis between the NSCLC cohort and the control group using the limma package (v3.10.3, http://www.bioconductor.org/packages/2.9/bioc/html/limma.html). The threshold was false discovery rate (FDR) < 0.05, and an absolute log2 fold change (|log2FC|) > 2. With “lipopolysaccharide” as the keyword, 6,571 LRGs were retrieved from the Comparative Toxicogenomics Database (CTD, http://ctdbase.org/), which are listed in Supplementary Table S3. The intersection of these LRGs with differentially expressed genes yielded a refined group of differentially expressed LRGs.

### Functional enrichment analysis of differentially expressed LRGs and identification of prognostic LRGs

The clusterProfiler package (v3.14.3) was used to conduct Gene Ontology (GO) and Kyoto Encyclopedia of Genes and Genomes (KEGG) pathway enrichment analyses of differentially expressed LRGs. Significant enrichment terms were selected using the threshold of a *p* < 0.05 and a count ≥2. By incorporating the expression of differentially expressed LRGs with survival data from NSCLC samples, we employed univariate Cox regression analysis using the survival package (v2.41–1, available at http://bioconductor.org/packages/survivalr/). Prognosis-associated LRGs were selected with a threshold of P < 0.01. We integrated prognosis-associated LRGs into the STRING database (v10.0, http://www.string-db.org/), with the protein-protein interaction (PPI) confidence score threshold set at 0.15. The resulting PPI network was visualized and mapped using Cytoscape software (v3.9.1).

### Unsupervised clustering analysis utilizing prognosis-related LRGs

Based on the expression profiles of prognosis-associated LRGs, unsupervised clustering of patients with NSCLC was performed using the Consensus ClusterPlus package (v1.54.0; https://www.bioconductor.org/packages/release/bioc/html/ConsensusClusterPlus.html). Using the delineated LRG molecular subtypes and their associated prognostic data, we generated Kaplan-Meier (KM) curves for each subtype using the survival package. A log-rank test was used to evaluate the survival disparities across subtypes. Correlations between LRG molecular subtypes and clinical phenotypes were evaluated using heat maps and chi-square tests.

### Characterization of immune infiltration across diverse molecular subtypes of NSCLC

To delve deeper into the variations in the tumor immune microenvironment across distinct NSCLC subtypes, we utilized the single-sample Gene Set Enrichment Analysis (ssGSEA) algorithm to compute the enrichment scores for 28 immune cell types. Subsequently, we conducted Wilcoxon rank-sum tests to ascertain the distributional variance of these immune cells among different subtypes. The stromal score, immune score, ESTIMATE score, and tumor purity of the NSCLC samples were quantified using the ESTIMATE algorithm, and the differences among the different subtypes were evaluated using the Wilcoxon rank-sum test.

### Characterization of immune checkpoint and HLA gene expression across distinct molecular subtypes and elucidation of underlying molecular mechanisms

The expression profiles of HLA family genes and immune checkpoint genes (ICGs) were extracted from NSCLC samples. The Wilcoxon rank-sum test was used to evaluate differential expression across various molecular subtypes. Based on the gene expression profiles of TCGA NSCLC specimens, the ssGSEA algorithm was employed to filter for KEGG signaling pathways that were significantly associated with molecular subtype classification within the Gene Set Enrichment Analysis (GSEA) database (http://software.broadinstitute.org/gsea/downloads.jsp). The threshold for selecting pathways was set at P < 0.05.

### Construction and validation of the prognostic LRGs scoring model

Integrating prognosis-related LRGs with the survival data of TCGA NSCLC samples, we performed survival regression analysis using the Least Absolute Shrinkage and Selection Operator (LASSO) algorithm within the lars package (v1.2, available at https://cran.r-project.org/web/packages/lars/index.html). A ten-fold cross-validation approach was used to select predictive genes. Subsequently, a prognostic risk score model was developed using the following formula:

Riskscore=∑βgene×Expgene

The coefficient βgene signifies the value ascertained for the gene from LASSO regression, and Expgene quantifies the gene’s relative expression level in the TCGA training set. To further substantiate the model’s precision, the risk scores for individual samples in both TCGA and GEO external validation cohorts were computed. Samples from these cohorts were stratified into high-risk (risk score ≥ median risk score) and low-risk (risk score < median risk score) groups. The association between risk stratification and actual survival outcomes was evaluated using KM curve analysis. Distribution differences in various clinical characteristics such as sex, tumor histology, tumor stage, and smoking history among the different risk groups were compared using the chi-square test. Correlation analyses were performed between the prognostic LRGs in the model and the levels of immune cell infiltration and ICG expression in the NSCLC samples using the correlation function (cor).

### Construction and validation of a nomogram risk-prediction model

Clinical information was extracted from the TCGA NSCLC samples to assess the prognostic LRGs scoring model. Utilizing both univariate and multivariate Cox regression analyses available in the Survminer package (v0.4.9, accessible at https://cran.rstudio.com/web/packages/survminer/index.html), The prognostic LRGs scoring model and clinical characteristics of TCGA NSCLC patients were scrutinized for independently prognostic factors. Using the RMS package (v6.7–0; available at https://cran.r-project.org/web/packages/rms/), a nomogram model was developed based on the independent prognostic factors. The pROC package (v1.18.0, accessible at https://cran.r-project.org/web/packages/pROC/) generated Receiver Operating Characteristic (ROC) curves to assess the predictive proficiency of the nomogram model, and calibration curves were constructed using the RMS package to examine the concordance between model predictions and actual clinical prognostic outcomes.

### The correlation between nomoscore and somatic mutations, tumor mutation burden (TMB), and immunological factors

Using the nomogram model to calculate the nomoscore for patients with NSCLC, patients were divided into high- and low-risk groups based on the median nomoscore. Utilizing the somatic mutation data from the TCGA database, we employed the maftools package (v2.14.0, accessible at https://bioconductor.org/packages/release/bioc/html/maftools.html) to quantify the mutation frequencies of the top 20 somatic mutation genes. Subsequently, we calculated the TMB for all NSCLC samples and compared the TMB distributions among the different nomo-risk stratifications. NSCLC samples were classified according to their risk scores and TMB values. Within each nomo-risk stratification, the samples were divided into high- and low-TMB groups based on the median TMB value. A KM curve analysis was conducted for the four cohorts.

### Assessment of predictive efficacy of nomoscore for chemotherapy drug sensitivity and immunotherapy responsiveness in patients with NSCLC

The sensitivity of NSCLC patients to 138 chemotherapeutic agents was evaluated using the Genomics of Drug Sensitivity in Cancer database (https://www.cancerrxgene.org/). The half-maximal inhibitory concentration (IC50) of each therapeutic agent was quantified using the pRRophetic package (https://github.com/paulgeeleher/pRRophetic), and differences across distinct risk strata were compared using the Wilcoxon rank-sum test. We performed Tumor Immune Dysfunction and Exclusion (TIDE) analysis (http://tide.dfci.harvard.edu/) to assess the responsiveness of patients with NSCLC to immunotherapy. Differences in immunotherapy responses among distinct risk groups were analyzed using the Wilcoxon rank-sum test. The GSE135222 and GSE126044 datasets were retrieved from the NCBI for Biotechnology Information GEO repository. Patients diagnosed with NSCLC with documented responses to immunotherapy and prognostic information were selected. The risk scores were computed for each sample. The participants were subsequently stratified into high- and low-risk categories based on their respective risk scores. The Wilcoxon test was used to assess the differences in risk scores between the immunotherapy-responsive and non-responsive groups. Additionally, KM curves and the log-rank test were used to evaluate the significance of prognostic disparities between the high-risk and low-risk cohorts.

### Analysis of the correlation between nomoscore and immune markers

The clinical data of patients with NSCLC treated with anti-PD-1/PD-L1 therapy were downloaded from the NCBI GEO database (GSE135222 and GSE126044). Patients with information on post-treatment drug responses and survival data were selected from the two datasets, and their risk scores were calculated. All samples were divided into high- and low-risk groups using the median risk score as the threshold. The Wilcoxon rank-sum test was used to assess differences in risk scores between the two risk groups. The KM survival curves and log-rank tests were used to compare the survival rates between the high- and low-risk cohorts. Concurrently, the Wilcoxon signed-rank test was used to assess the differential expression of CD8A and PD-L1 between high- and low-risk cohorts. Following the CYT score calculation method (Rooney et al., 2015), we computed the cytolytic activity score (CYT) for NSCLC patients in TCGA database using the TPM values of the GZMA and PRF1 genes calculated using the geometric mean. We used the Wilcoxon rank-sum test to assess the differences between the high- and low-risk groups. Using the expression profiles of tertiary lymphoid structure (TLS) genes (Cabrita et al., 2020), we estimated the TLS score for each sample using the ssGSEA algorithm and analyzed the differences between various risk groups using the Wilcoxon test. Additionally, we performed correlation analyses to explore the relationship between the risk scores and immune markers.

## Results

### Identification of differentially expressed prognostic LRGs

**FIGURE 1:**
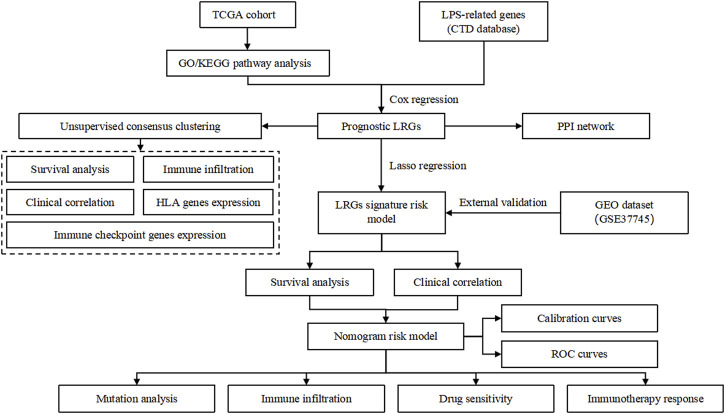
Flowchart of the study.

**FIGURE 2:**
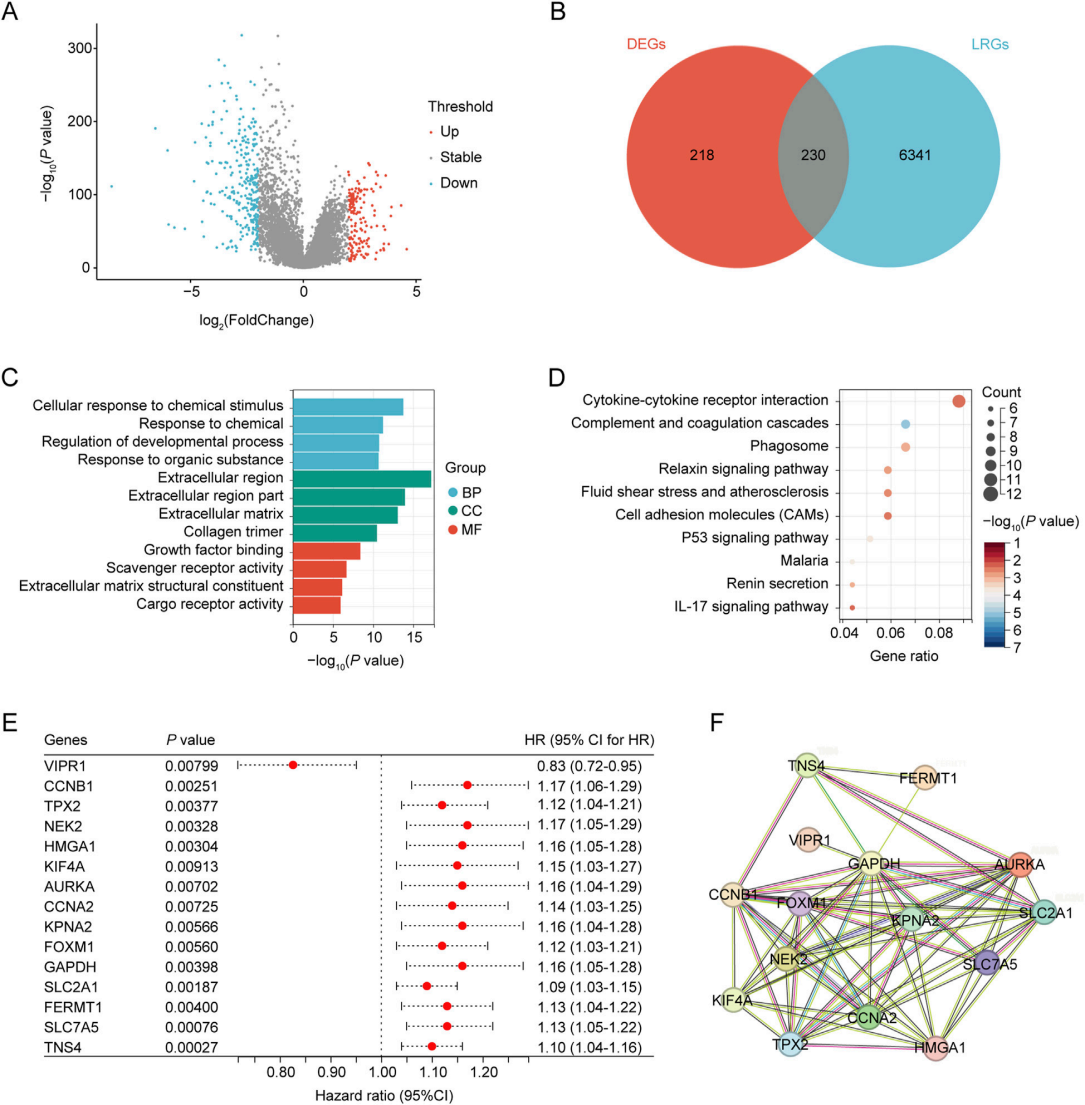
Identification of differentially expressed ARGs. **(A)** Volcano plot showing differentially expressed genes (DEGs) between the NSCLC and control groups. **(B)** Venn diagram, T vs. N DEGs in red, and LPS-related genes obtained from CTD in blue. **(C)** GO analysis of identified LRGs. **(D)** KEGG analysis of the identified LRGs. **(E)** LRGs showing significant prognostic correlation. **(F)** PPI network of the prognostic LRGs.

The workflow of this study is illustrated in Figure 1. In total, 448 differentially expressed genes were identified between NSCLC samples and normal lung tissues from the TCGA dataset. This set comprised 164 upregulated and 284 downregulated genes, as shown in Figure 2A and Supplementary Table S4. Meanwhile, 6,571 LRGs were retrieved from the CTD database (Supplementary Table S3). Upon intersecting the differentially expressed genes with the LRGs, we identified 230 LRGs that exhibited differential expression (Figure 2B and Supplementary Table S5). Functional enrichment analysis indicated that the functions of the differentially expressed LRGs predominantly pertained to responses to chemicals and chemical stimuli, interactions between cytokines and cytokine receptors, and inflammatory responses (Figures 2C, D and Supplementary Table S6). Univariate Cox regression analysis of the differentially expressed LRGs further identified 15 LRGs with significant prognostic correlations (P < 0.01) (Figure 2E). Subsequently, a PPI network comprising 15 nodes and 66 edges was constructed using prognostic LRGs (Figure 2F).

### Molecular clustering based on the prognostic LRGs

**FIGURE 3:**
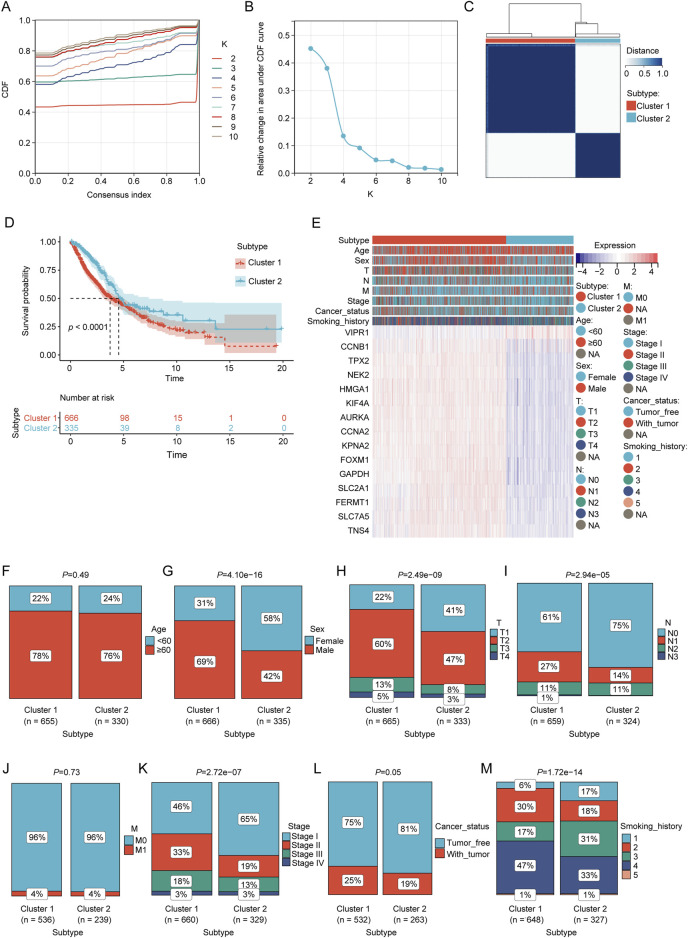
Consensus clustering analysis of prognostic LRGs. **(A, B)** Consensus clustering cumulative distribution function (CDF) with k values ranging from 2 to 10. **(C)** Consensus matrix heat map defining two clusters (k = 2). **(D)** Kaplan–Meier curves of the two clusters. **(E)** Differences in clinical characteristics and LRG expression between the two clusters. Within the classification of tumor status, “tumor-free” denotes a condition in which the patient remains without any tumor lesions up until the point of follow-up. This status encompasses scenarios post-surgery where the tumor has been successfully removed and no new tumor lesions have been identified, and “with tumor” indicates the emergence of new tumor growths following surgical intervention. **(F–M)** Comparison of clinical characteristics between clusters 1 and 2.

Based on the prognostic LRG expression and clinical outcomes, unsupervised clustering analysis was performed on the NSCLC datasets. The optimal number of clusters (k = 2) was deduced from the Cumulative Distribution Function (CDF) curves (Figures 3A, B). The results of clustering are presented in Figure 3C, where patients were categorized into two distinct clusters: Cluster 1 (n = 666) and Cluster 2 (n = 335). KM analysis revealed that Cluster 1 was associated with worse overall survival (OS) outcomes than Cluster 2 (Figure 3D). The heatmap elucidates the associations between the expression profiles of these prognostic LRGs and pertinent clinical parameters, including cluster subtype classification, age, sex, tumor staging, and documented smoking history (Figure 3E). Chi-square test revealed a significant correlation between the molecular subtypes of LPS and several clinical variables, including sex, tumor size (T), nodal involvement (N), overall stage, cancer status, and smoking history (*p* < 0.01; Figures 3F–M).

### Characterization of immune infiltration across distinct molecular clusters

We compared the immune cell abundances and immune stromal scores of clusters 1 and 2 to elucidate the differences in their immune infiltration. ssGSEA demonstrated significant differences in 26 immune cell types between the two clusters (*p* < 0.05, Supplementary Figure S1A). Notably, T cells, B cells, NK cells, monocytes, macrophages, and mast cells displayed significant disparities between the two subclusters. ESTIMATE analysis demonstrated that Cluster 1 exhibited significantly reduced ESTIMATE scores, ImmuneScores, and StromalScores compared to Cluster 2 (*p* < 0.05; Supplementary Figure S1B–D). The tumor purity of cluster 1 was significantly higher than that of cluster 2 (*p* < 0.05; Supplementary Figure S1E). Additionally, 10 ICGs (CD274, CTLA4, ICOS, HAVCR2, LAG3, CD47, BTLA, SIRPA, TNFRSF4, and VTCN1) exhibited significant differential expression between the two subclusters (*p* < 0.05, Supplementary Figure S1F). Similarly, a notable difference was observed in the expression levels of the HLA gene family between the two subclusters (*p* < 0.05; Supplementary Figure S1G).

### Establishment and verification of prognostic LRGs scoring model

**FIGURE 4:**
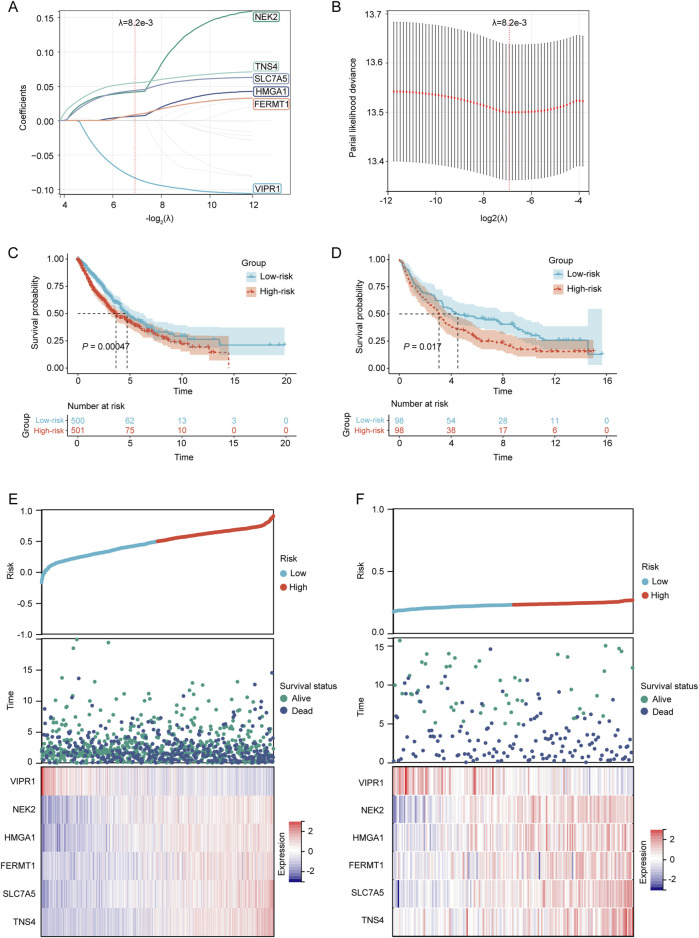
Establishment and verification of the prognostic LRGs scoring model. **(A)** LASSO coefficient profile of the LRGs. **(B)** Selection of the optimal parameter (λ.min) in the LASSO model. **(C–D)** KM survival curves for patients in the high/low-risk groups within the TCGA training cohort and GEO validation cohort, respectively. **(E–F)** Distribution of risk scores, overall survival times, and expression patterns of model genes across TCGA training cohort and GEO validation cohort.

To construct a prognostic model for patients with NSCLC based on LRGs, univariate Cox regression analysis and subsequent LASSO regression were used to select the optimal combination of genes, yielding a set of six genes, namely, VIPR1, NEK2, HMGA1, FERMT1, SLC7A5, and TNS4 (Figures 4A, B). The regression coefficient of these LRGs was −0.0836904381433785, 0.0414727838593598, 0.00620029806120873, 0.00831582925035488, 0.0437362956589142, and 0.0546254823173021, respectively. Therefore, the predictive risk score model was developed as follows:

$$\text{Riskscore}=\text{VIPR}1*\left(-0.0836904381433785\right)+\text{NEK}2*0.0414727838593598+\text{HMGA}1*0.00620029806120873+\text{FERMT}1*0.00831582925035488+\text{SLC}7A5*0.0437362956589142+\text{TNS}4*0.0546254823173021$$

Based on the median risk score, NSCLC patients in both the TCGA training cohort and the GEO validation cohort were stratified into high-risk and low-risk groups. The KM survival curves demonstrated that patients in the high-risk category had a poorer prognosis than those in the low-risk group in both cohorts (*p* < 0.05) (Figures 4C, D). The prognostic heat maps that encapsulated the risk score, distribution of survival times, and expression profiles of model genes facilitated the delineation of the association between gene expression patterns and patient survival status in both cohorts (Figures 4E, F).

### Correlation between risk score and clinical characteristics of NSCLC patients

**FIGURE 5:**
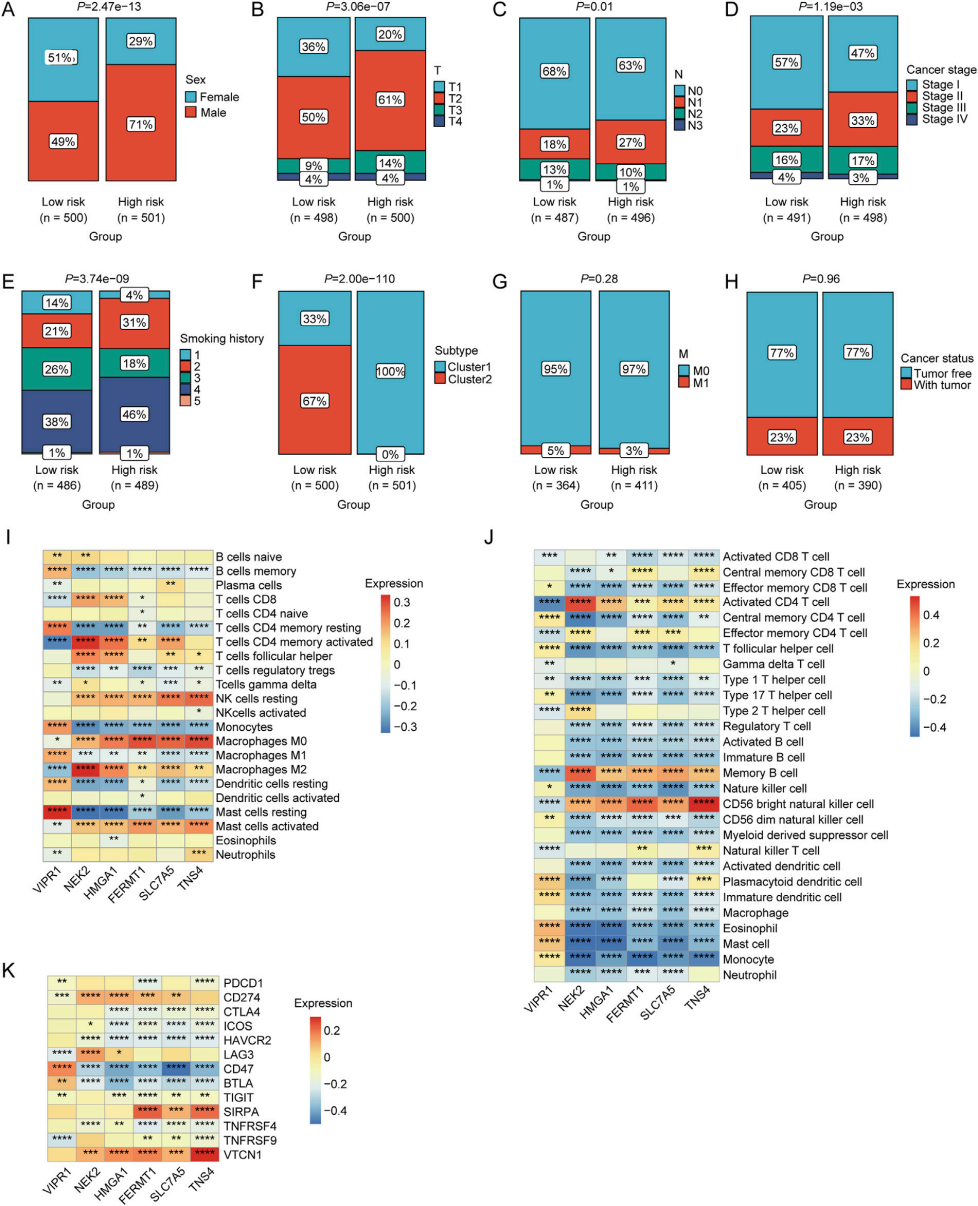
Associations between risk scores and various clinical characteristics. Correlation of LRGs score with **(A)** sex, **(B)** T stage, **(C)** N stage, **(D)** clinical stage, **(E)** smoking history, **(F)** molecular clusters, **(G)** M stage, and **(H)** cancer status. **(I)** Correlation between the expression levels of immune cells and prognostic LRGs determined using CIBERSORT analysis. **(J)** Correlation between the expression levels of immune cells and prognostic LRGs, as determined by ssGSEA. **(K)** Correlation between immune checkpoint genes and prognostic LRGs.

The chi-square test revealed a significantly higher proportion of male patients in the high-risk group than in the low-risk group (*p* < 0.05; Figure 5A). Patients in the high-risk group exhibited higher T-stage-, N-, and cancer-stage distribution characteristics (*p* < 0.05, Figures 5B–D). Furthermore, the high-risk group had a higher prevalence of patients with a longer smoking history (*p* < 0.05; Figure 5E). The proportion of patients in Cluster 1 was significantly higher in the high-risk group (*p* < 0.05; Figure 5F). However, no significant differences were observed between the two risk groups in terms of M stage or tumor status classification (*p* < 0.05, Figures 5G, H). Moreover, a strong correlation was observed between the relative abundance of macrophages and mast cells and the expression levels of LRGs in the model (VIPR1, NEK2, HMGA1, FERMT1, SLC7A5, and TNS4) (Figure 5I). Significant correlations were observed between the expression of these model LRGs and the infiltration of multiple immune cells such as activated CD4 + T cells, CD56 bright natural killer cells, macrophages, mast cells, and monocytes (Figure 5J). Furthermore, multiple ICGs were associated with these model LRGs (Figure 5K).

### Incorporation of the prognostic LRGs scoring model as an independent predictor in nomogram model construction

**FIGURE 6:**
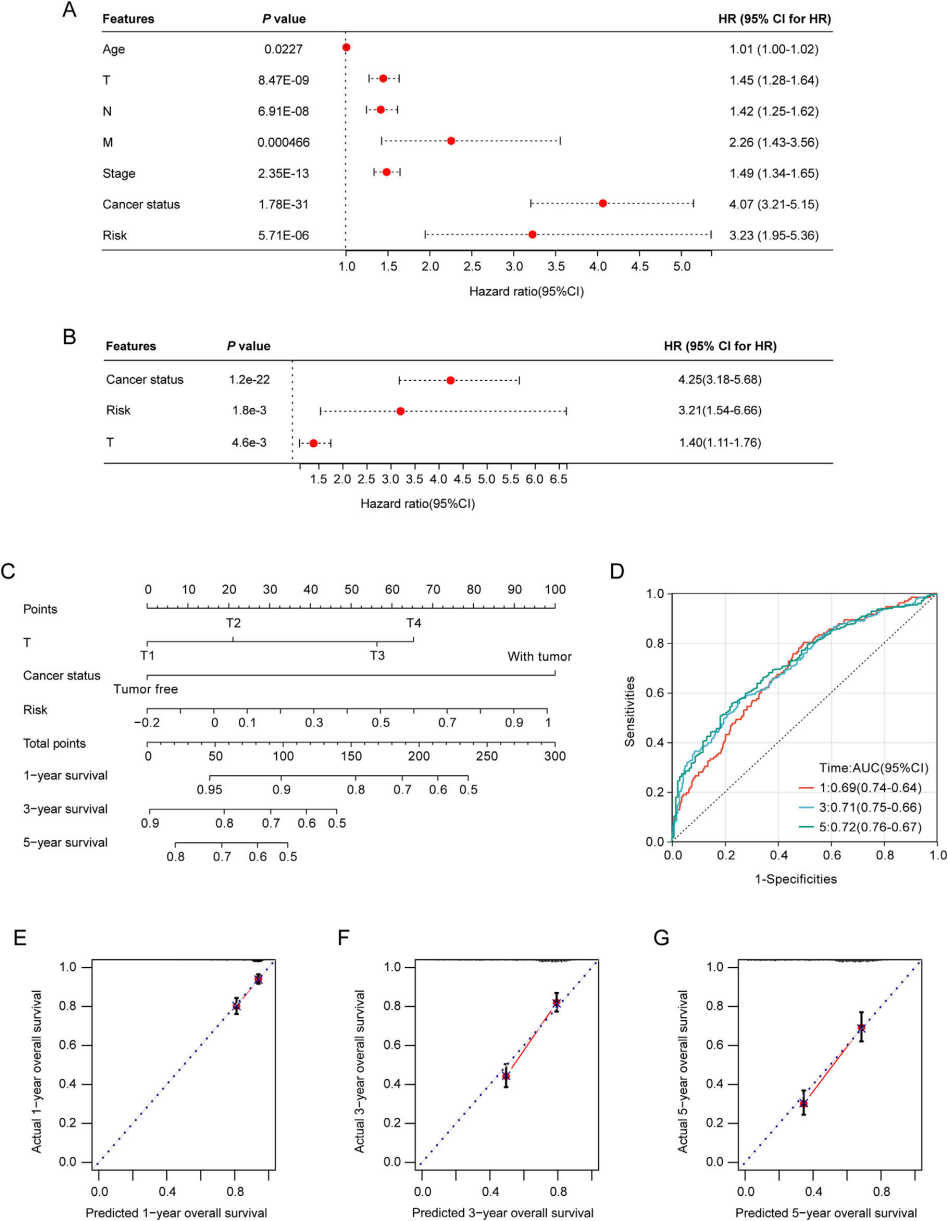
Construction and validation of the nomogram model. **(A)** Association of patient age, clinical tumor stage, TNM stage, and risk score with the prognosis of NSCLC patients. **(B)** Relationship between tumor stage, T-stage, risk score, and prognosis of NSCLC patients. **(C)** Nomogram model for predicting clinical outcomes of patients with NSCLC. **(D)** Area under the receiver operating characteristic (ROC) curves (AUCs) for the nomogram model’s predictive performance at 1-, 2-, and 3-year intervals. **(E–G)** Calibration curves were used to evaluate the predictive accuracy of the nomogram model at 1-, 2-, and 3-year benchmarks.

To rigorously evaluate the prognostic value of the LRGs scoring model, both univariate and multivariate Cox regression analyses revealed that tumor stage, T stage, and risk score served as independent prognostic predictors of the outcomes of patients with NSCLC (Figures 6A, B). The identified independent prognostic factors were integrated into a predictive nomogram model, which facilitated the prediction of the clinical prognosis in patients with NSCLC (Figure 6C). The ROC analysis indicated that the nomogram model achieved area under the curve (AUCs) of 0.69, 0.71, and 0.72 for the 1-year, 3-year, and 5-year predictions, respectively (Figure 6D). Calibration curves also demonstrated good predictive accuracy for 1-year, 3-year, and 5-year outcomes (Figures 6E–G).

### Variations in somatic cell mutations, drug sensitivity and immunotherapy response among NSCLC patients in diverse risk categories

**FIGURE 7:**
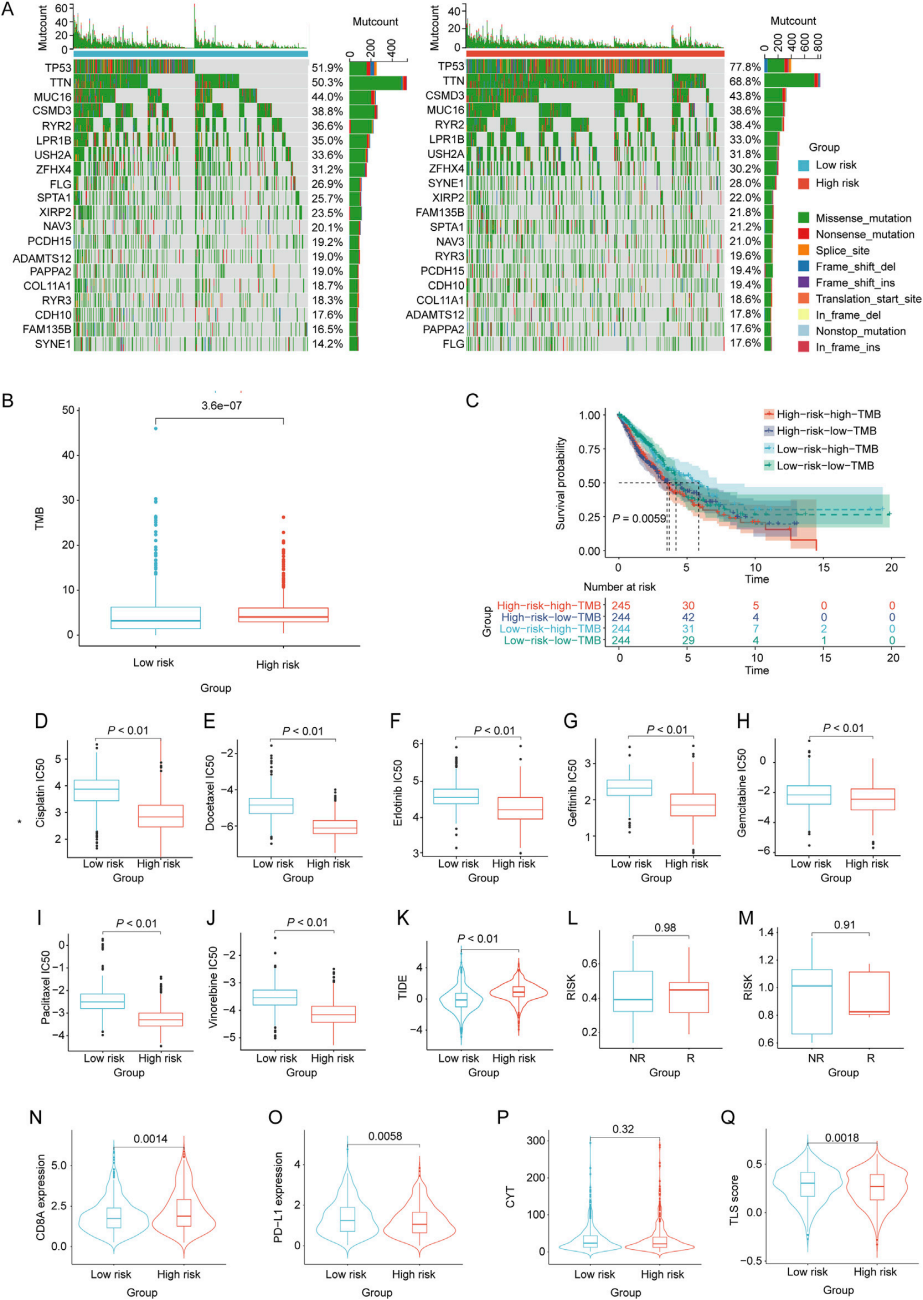
Evaluation of tumor mutation burden, immunotherapy response, and drug sensitivity based on the nomoscore. **(A)** Waterfall plots of the mutation distribution of the top 20 most frequently mutated genes in NSCLC patients from the low- and high-risk groups. **(B)** TMB between the low- and high-risk groups. **(C)** KM curves of NSCLC samples from the low-risk low-TMB, low-risk high-TMB, high-risk low-TMB, and high-risk high-TMB groups. **(D–J)** IC50 values of different drugs were compared between the high- and low-risk groups. **(K)** Differences in TIDE between high- and low-risk groups. **(L, M)** Differences in risk scores between the high-risk and low-risk groups in the GSE135222 and GSE126044 datasets. Violin plots indicating differences in **(N)** CD8A values, **(O)** PD-L1 values, **(P)** CYT scores, and **(Q)** TLS scores between the high- and low-risk groups.

By utilizing the nomogram model, the nomoscore for patients can was calculated, and patients were categorized into high- and low-risk groups. We then identified the frequencies of the top 20 somatic cell variations in the nomoscore risk rating groups. The result revealed that the frequencies of the top 20 mutations were slightly higher in the high-risk group than in the low-risk group (Figure 7A). Additionally, we comprehensively assessed the TMB levels in each NSCLC sample, revealing that the high-risk group exhibited significantly elevated TMB levels (*p* < 0.05, Figure 7B). NSCLC samples were divided into high- and low-TMB groups based on the median TMB. KM curve analysis revealed a significant association between adverse prognosis and the high-risk high-TMB group in patients with NSCLC (Figure 7C). To evaluate further the correlation between the immune landscape of NSCLC and prognostic LRGs, we analyzed the differences in immune cell abundance and immune stromal scores between the two groups. Significant differences in the abundance of 15 immune cell types between the two groups were detected using CIBERSORT analysis (*p* < 0.05). Notably, the high-risk group exhibited a marked increase in the number of macrophages, mast cells, and dendritic cells (Supplementary Figure S2A). Moreover, the high-risk group exhibited a significant increase in the number of activated CD4 + T and CD56 bright natural killer cells, as revealed by ssGSEA (Supplementary Figure S2B).

To assess chemotherapeutic drug sensitivity, the IC50 values of 138 chemotherapeutic agents were calculated (Supplementary Table S7). In the high-risk group, we observed significantly lower IC50 values for commonly used chemotherapy drugs, including BI.2536, Bosutinib, CGP.082996, Docetaxel, RO.3306, and vinblastine, than in the low-risk group (*p* < 0.01, Figures 7D–J). To evaluate the response of patients with NSCLC to immune therapy, TIDE analysis revealed a significant correlation between higher TIDE scores and a diminished response to immunotherapy in NSCLC patients classified as high-risk (*p* < 0.05, Figure 7K). Moreover, the GSE135222 and GSE126044 datasets, which contain data on patients with NSCLC treated with anti-PD-1/PD-L1 therapy, were selected to analyze the immunotherapy response. There was no significant difference in the risk scores between the immunotherapy response (R) and non-response (NR) groups in either dataset (Figures 7L, M). Furthermore, the results showed that the CD8A levels in the high-risk group were significantly higher than those in the low-risk group (*p* < 0.05). Conversely, the PD-L1 and TLS scores in the high-risk group were significantly lower than those in the low-risk group (*p* < 0.05). However, there was no significant difference in the CYT scores between the two groups (Figures 7N–Q).

## Discussion

A strong correlation exists between the lung and gut microbiota and NSCLC progression. Specifically, the introduction of bacteria isolated from late-stage lung cancer into the trachea significantly accelerates tumor growth (Jin et al., 2019). A study conducted in patients with lung cancer revealed that the composition of the gut microbiota is associated with TNM staging and primary tumor size (Otoshi et al., 2022). Zhang et al. revealed that the colonization of lung cancer lesions predominantly consists of gram-negative bacteria (Zhang WQ. et al., 2018). Furthermore, the gut microbiota of patients with lung cancer exhibit elevated levels of Proteobacteria (Qin et al., 2014). Lu et al. revealed a significant association between dysbiosis of the gut and sputum microbiota and progression and distant metastasis (DM) in NSCLC (Lu et al., 2021). Similarly, a study focusing on patients with advanced lung cancer (stage IIIB, IV) and metastasis observed an elevated presence of *Thermus* and *Legionella* within lung cancer lesions (Yu et al., 2016). Microorganisms residing in tumor lesions or the intestines of patients with NSCLC may participate in the progression of lung tumors via various mechanisms. For instance, Bacteroidetes can inhibit tumor proliferation by upregulating T-cell levels within the tumor microenvironment, whereas Firmicutes can activate T-regulatory cells and facilitate cancer progression (Pizzo et al., 2022). Microorganisms modulate the progression of lung cancer by producing enzymatically active toxins. For instance, *Bacteroides fragilis* toxin, *E. coli* Cif, cytotoxic necrotizing factor 1 (CNF1), *Fusobacterium nucleatum* FadA, and *Salmonella* AvrA can induce the development and progression of lung cancer by participating in the relevant signaling pathways (Fiorentini et al., 2020). Furthermore, the involvement of *Helicobacter pylori* in lung cancer development has been observed primarily through the cytotoxicity of its main protein toxin VacA and its ability to promote the secretion of the pro-inflammatory cytokines IL-6 and IL-8 (Nakashima et al., 2015).

LPS induced inflammatory damage in the pulmonary system. LPS can also induce localized inflammation and facilitate cancer progression by triggering the release of pro-inflammatory cytokines from cancer cells (Xu et al., 2019). Additionally, it activates TLR4, enhancing the expression of NLRP3 and promoting the secretion of chemotactic factors and inflammatory mediators from the cancer cells. This cascade increases SPP1 expression, facilitating the binding of transglutaminase to the extracellular matrix, thereby mediating tumor cell adhesion and fostering malignant biological activity (Zhang et al., 2023). However, the precise mechanism by which LPS affects NSCLC has not yet been fully elucidated. This study identified LRG-based NSCLC clusters with distinct molecular and cellular profiles and various prognoses. Through the integration of functional enrichment and immune infiltration analyses of differentially expressed LRGs, we explored the molecular mechanisms by which gram-negative bacteria participate in NSCLC via LPS. Additionally, we established a prognostic risk model and nomogram. Importantly, our findings provide an overall understanding of the significance of the microbiome and its components in NSCLC progression.

In this study, a prognostic risk-scoring model for NSCLC was established based on prognostic LRGs that were significantly differentially expressed between NSCLC and control groups (VIPR1, NEK2, HMGA1, FERMT1, SLC7A5, and TNS4). Among these, VIPR1 has been identified as a protective gene for the survival of NSCLC patients, whereas NEK2, HMGA1, FERMT1, SLC7A5, and TNS4 are risk genes. VIPR1 serves as a receptor for the vasoactive intestinal peptide (VIP), a neuropeptide known for its anti-inflammatory and immunomodulatory properties. Studies have demonstrated that upon stimulation with LPS, the activation of VIPR1 can effectively suppress inflammatory responses, inhibit the release of inflammatory mediators, diminish cellular and DNA damage resulting from inflammation, and exert a restraining effect on tumor development and progression (Delgado and Ganea, 2013). Nek2 is an oncogene highly expressed in multiple cancers, including breast cancer. Its overexpression promotes cancer cell proliferation and enhances drug resistance (Cappello et al., 2014; Zhou et al., 2013). Moreover, upon LPS stimulation, NEK2 overexpression plays a crucial role in tumor metastasis and invasion by initiating and promoting epithelial-mesenchymal transition (EMT) (Zhang Y. et al., 2018). Additionally, NEK2 regulates cell cycle progression by promoting cell entry into mitosis and facilitating cellular self-renewal, thus increasing the likelihood of cancer development (Lin et al., 2016). Currently, there is a shortage of research on the stimulatory effects of LPS on HMGA1 expression. As a transcription factor, HMGA1 activates the expression of numerous cancer-promoting genes while inhibiting the expression of various apoptosis-related genes, thus enhancing cancer cell resistance to apoptosis (Fusco and Fedele, 2007). Furthermore, HMGA1 facilitates EMT and tumor metastasis in cancer (Shah et al., 2013). FERMT1 promotes cell migration and infiltration by binding to integrins in the extracellular matrix, thereby promoting the metastasis and dissemination of cancer cells (Sossey-Alaoui et al., 2014). In addition, it contributes to cancer cell proliferation and growth by regulating the cell cycle (Rognoni et al., 2014). SLC7A5 facilitates cancer initiation and progression by mediating the transport of amino acids and modulating the mTOR signaling pathway (Yanagida et al., 2001).

Numerous studies have shown that intratumoral immune infiltrates are associated with the clinical outcomes of NSCLC and can predict the response to immunotherapy (Federico et al., 2022; Tian et al., 2023). The pro-tumor immune environment is characterized by a decrease in cytotoxic CD8^+^ T cells and NK cells and an increase in exhausted CD8^+^ T cells, immunosuppressive CD4^+^ FOXP3+ Tregs, regulatory B cells, CD4^+^ T cells with a pro-inflammatory Th2 phenotype, and abundant M1-like macrophages and neutrophils (TANs) (Federico et al., 2022). In our study, we observed a decrease in CD8^+^ T cells, NK cells, and DCs, along with an increase in CD4^+^ T cells in cluster 1, which was characterized by higher expression of risk genes and poorer prognosis. Moreover, VIPR1, as a protective factor, was positively associated with M1-like macrophages and monocytes but negatively correlated with mast cells and M2-like macrophages. In contrast, NEK2, HMGA1, FERMT1, SLC7A5, and TNS4 showed the opposite correlations with these immune cells. Therefore, LRGs may influence NSCLC progression by shaping the immune landscape of the disease.

A risk stratification model based on these prognostic LRGs was constructed to evaluate the effects of LPS-associated genetic alterations on the survival of patients with NSCLC. KM survival analyses revealed that patients diagnosed with NSCLC with elevated risk scores had suboptimal prognostic attributes and diminished lifespans. Additionally, a low-risk score was associated with favorable clinical characteristics, such as earlier tumor staging in patients with NSCLC. Moreover, by integrating the risk scores with selected clinical parameters, we constructed a nomogram model. The calibration curve and ROC analysis confirmed that the nomogram model exhibited high predictive precision.

Selecting patients who are most likely to benefit from ICIs is crucial for increasing the efficacy of ICI and minimizing ICI-related adverse events in patients with NSCLC. Our findings revealed a significant positive correlation between the nomoscore and CD8A expression and a negative correlation between PD-L1 expression and the TLS score. The TIDE algorithm has been utilized extensively to evaluate immune responses to ICIs in a multitude of research studies. Our nomoscore exhibited a trend parallel to that of the TIDE predictor in predicting immunotherapy response. These results validate the potential of the nomoscore as a robust prognostic indicator of immunotherapeutic outcomes in patients with NSCLC.

Our findings offer a new understanding of the influence and underlying molecular mechanisms of the microbiota on NSCLC survival and the immune environment. Further clinical and experimental studies are required to validate these findings. However, the LPS producing gram-negative bacteria including *Escherichia-shigella* and *Klebsiella* have been confirmed to exacerbate the lung injury (Sun et al., 2020). *Klebsiella* is capable of disrupting the gut barrier, eliciting systemic or localized inflammatory responses that disrupt the host’s overall immune balance, thereby worsening inflammatory lung injury (Belkaid and Hand, 2014). Other gram-negative bacteria, *such as Pseudomonas aeruginosa*, *Escherichia coli*, *Klebsiella pneumoniae*, or *Acinetobacter baumannii*, are also identified as major causative pathogens of different respiratory tract infections (Rodrigo-Troyano and Sibila, 2017). During gram-negative bacterial infections, LPS can activate host immune cells, triggering an inflammatory response (Abdulnour et al., 2016). LPS modification in gram-negative bacteria during chronic infection could influence the gene expression profiles, possibly contributing to infection establishment and progression (Maldonado et al., 2016). Therefore, the LPS signature is likely to originate from pulmonary infections or exacerbations rather than the gut microbiome, and there is insufficient evidence to indicate that LPS related to the gut-lung axis takes precedence over pulmonary infections in generating LPS-related gene signatures. Future research could focus on investigating the distinct roles of pulmonary infections and the gut microbiome in generating LPS-related gene signatures to better understand NSCLC pathogenesis and develop potential therapeutic targets. Additionally, the existing animal and cellular models do not precisely replicate the nuanced microbiome milieu of NSCLC. Currently, investigations into the contribution of the microbiome to NSCLC pathophysiology are still in the preliminary phase, bringing an emerging potential for utilizing the microbiome not only as a diagnostic instrument but also as a novel immunotherapeutic modality for NSCLC.

## Data Availability

The original contributions presented in the study are included in the article/Supplementary Material, further inquiries can be directed to the corresponding authors.

## Supplementary material

SUPPLEMENTARY TABLE S1 Clinical information of NSCLC patients in TCGA dataset.

SUPPLEMENTARY TABLE S2 Clinical information of NSCLC patients in the GEO dataset (GSE37745).

SUPPLEMENTARY TABLE S3 LRGs were downloaded from the CTD database.

SUPPLEMENTARY TABLE S4 Differentially expressed genes between the NSCLC and control groups.

SUPPLEMENTARY TABLE S5 LRGs were differentially expressed between the NSCLC and control groups.

SUPPLEMENTARY TABLE S6 Enrichment analysis of differentially expressed LRGs in the KEGG/GO pathways.

SUPPLEMENTARY TABLE S7 Statistical differences in IC50s between high-risk- and low-risk groups.

SUPPLEMENTARY FIGURE S1 Differential immune infiltration characteristics between clusters 1 and 2. **(A)** Differential expression levels of immune cells between clusters 1 and 2 as indicated by ssGSEA. **(B–E)** Differences in ESTIMATE score, ImmuneScore, StromalScore, and tumor purity between Clusters 1 and 2. **(F)** Expression levels of ICGs in clusters 1 and 2. **(G)** Expression levels of the HLA genes in clusters 1 and 2.

SUPPLEMENTARY FIGURE S2 Differences in immune cell expression levels between high- and low-risk groups were detected using **(A)** CIBERSORT and **(B)** ssGSEA.

## Glossary

NSCLC: non-small cell lung cancer
LRGs: lipopolysaccharide-related genes
LASSO: Least Absolute Shrinkage and Selection Operator
KM: Kaplan–Meier
TME: tumor microenvironment
GLA: gut-lung axis
LPS: lipopolysaccharide
TLR: toll-like receptor
TCGA: The Cancer Genome Atlas
GEO: Gene Expression Omnibus
CTD: Comparative Toxicogenomics Database
GO: gene ontology
KEGG: Kyoto Encyclopedia of Genes and Genomes
PPI: protein-protein interaction
ssGSEA: single-sample Gene Set Enrichment Analysis
ICGs: immune checkpoint genes
GSEA: Gene Set Enrichment Analysis
ROC: receiver operating characteristic
TMB: tumor mutation burden
IC50: half-maximal inhibitory concentration.
TIDE: tumor immune dysfunction, and exclusion
CYT: cytolytic activity score
TLS: Tertiary lymphoid structure
CDF: cumulative distribution function
AUC: area under the curve
BM: brain metastasis
CDT: cytolethal-distending toxin
CNF1: Cytotoxic necrotizing factor 1
VEGF: Vascular endothelial growth factor
VIP: vasoactive intestinal peptide
BMDCs: bone marrow-derived dendritic cells
TAAs: tumor-associated antigens
IFN: interferon
SCF: stem cell factor
Tregs: T cells
MHC: major histocompatibility complex
ULBPs: UL16-binding proteins
IrAEs: immune-related adverse events
