# Supplementary material for: A scoring model based on bacterial lipopolysaccharide-related genes to predict prognosis in NSCLC
Source: Front Genet. 2024 Nov 14;15:1408000. doi: 10.3389/fgene.2024.1408000 (PMC11602480; doi:10.3389/fgene.2024.1408000)
Supplement: Supplementary file 3 [file Table8.docx]

1. Limma for screening differentially expressed genes

library(limma)

data<-read.table("input.txt",sep = "\t",header=T,row.names=1)

data<- as.matrix(data)

mode(data)

group<-read.table("group.txt",sep = "\t",header=T,row.names=1)

design <- model.matrix(~ -1+factor(group))

colnames(design)<-c("Normal","Tumor")

design

contrast.matrix <- makeContrasts(Tumor-Control,levels=design)

fit <- lmFit(data, design)

fit1 <- contrasts.fit(fit, contrast.matrix)

fit2 <- eBayes(fit1)

dif <- topTable(fit2, coef = 1, n = nrow(fit2), lfc = 0)

write.table(dif, "allgene.dif.txt", row.names = TRUE, sep = "\t")

write.csv(dif,"allgene.dif.csv")

2. GO and KEGG enrichment analysis

library(clusterProfiler)

degenes <- read.csv('input.txt',header = T,stringsAsFactors = F,sep = '\t')

genelist <- degenes$Entrez.ID

genelist[duplicated(genelist)]

go <- enrichGO(genelist, OrgDb = org.Hs.eg.db, ont='ALL',pAdjustMethod = 'BH',pvalueCutoff = 0.05,

qvalueCutoff = 0.2,keyType = 'ENTREZID')

barplot(go,showCategory=20,drop=T)

dotplot(go,showCategory=50)

kegg <- enrichKEGG(genelist, organism = 'hsa', keyType = 'kegg', pvalueCutoff = 0.05,pAdjustMethod = 'BH',

minGSSize = 10,maxGSSize = 500,qvalueCutoff = 0.2,use_internal_data = FALSE)

dotplot(kegg, showCategory=30)

3. Univariate Cox regression analysis

library("survival")

library("survminer")

data=read.table("data.txt",header = T,row.names = 1,sep = "\t")

res.cox <- coxph(Surv(time, status) ~ FDX1, data = data)

covariates<-colnames(data)[3:500]

univ_formulas <- sapply(covariates,

function(x) as.formula(paste('Surv(time, status)~', x)))

univ_models <- lapply( univ_formulas, function(x){coxph(x, data = data)})

univ_results <- lapply(univ_models,

function(x){

x <- summary(x)

p.value<-signif(x$wald["pvalue"], digits=2)

HR <-signif(x$coef[2], digits=2);

HR.confint.lower <- signif(x$conf.int[,"lower .95"], 2)

HR.confint.upper <- signif(x$conf.int[,"upper .95"],2)

Hazard_Ratio <- paste0(HR, " (",

HR.confint.lower,"-",HR.confint.upper, ")")

res<-c(p.value,HR,HR.confint.lower,HR.confint.upper,Hazard_Ratio)

names(res)<-c("p.value","HR","lower","upper","HR (95% CI for HR)")

return(res)

})

res <- t(as.data.frame(univ_results, check.names = FALSE))

result=as.data.frame(res)

View(result)

write.table(result, "result.txt",row.names = T,col.names = T,sep="\t")

4. Subtype analysis

library(ConsensusClusterPlus)

A=read.table("input.txt",header=T)

d=data.matrix(A[,-1])

title=tempdir("Documents and Settings\Administrator")

results = ConsensusClusterPlus(d,maxK=6,reps=50,pItem=0.8,pFeature=1,title=title,clusterAlg="km",distance="euclidean",seed=1262118388.71279,plot="png")

5. Survival curve analysis

library(survival)

library(survminer)

data=read.table("data.txt",header = T,row.names = 1,sep = "\t")

fit<- survfit(Surv(time, status) ~ group,data = data)

ggsurvplot(fit,data=data,pval = TRUE, conf.int = TRUE, title = "Survival curves", linetype = "strata", risk.table = TRUE, legend.title ="group",legend.labs = c("H", "L"),risk.table.col = "strata",surv.median.line = "hv", palette = c("red","blue"))

6. Immune infiltration

library(data.table)

setwd("C:/Users/Administrator/Desktop")

DataDirectory<-"C:/Users/Administrator/Desktop/"

if(T){

rawdata_file<-"DATA.txt"

raw_data <- fread(rawdata_file, sep = '\t', header = T,stringsAsFactors=FALSE)

raw_data <- as.data.frame(raw_data)

raw_data[1:4,1:4]

}

if(T){

rownames(raw_data) <- raw_data[,1]

raw_data <- raw_data[,-1]

raw_data <- 2^raw_data-1

raw_data[1:4,1:4]

}

if(T){

countToTpm <- function(counts, effLen)

{

rate <- log(counts) - log(effLen)

denom <- log(sum(exp(rate)))

exp(rate - denom + log(1e6))

}

countToFpkm <- function(counts, effLen)

{

N <- sum(counts)

exp( log(counts) + log(1e9) - log(effLen) - log(N) )

}

fpkmToTpm <- function(fpkm)

{

exp(log(fpkm) - log(sum(fpkm)) + log(1e6))

}

countToEffCounts <- function(counts, len, effLen)

{

counts * (len / effLen)

}

}

if(T){

TPM_expr <- as.data.frame (apply(raw_data , 2, fpkmToTpm))

TPM_expr[1:4,1:4]

rm(raw_data)

all(floor(colSums(TPM_expr))>999998 &&ceiling(colSums(TPM_expr))<1000002)

}

if(T){

TPM_expr_file <- paste0(DataDirectory, "DATA.","tpm",".tsv")

write.table(TPM_expr, file =

TPM_expr_file, sep="\t", row.names =T,col.names =T, quote = F)#

}

library("gplots")

library("data.table")

library("dplyr")

library("ggplot2")

library("GenVisR")

library("descriptr")

library("vioplot")

library("pheatmap")

library("survival")

source("CIBERSORT.R"

if(T){

rawdata_file<-"DATA.tpm.tsv"

cibersort_file <- paste0("DATA.","cibersort",".txt")

results=CIBERSORT("LM22.txt", rawdata_file,

perm=100, QN=FALSE,cibersort_file)

print("CIBERSORT DONE!")

}

geneSet <- read.csv("ssGSEAcankao.txt",header = F,sep = "\t",) # downloded from http://cis.hku.hk/TISIDB/download.php

view(geneSet)

class(geneSet)

geneSet=t(geneSet)

colnames(geneSet)=geneSet[1,]

geneSet=geneSet[-1,]

a <- geneSet

a <- a[1:nrow(a),]

View(a)

set <- colnames(a)

l <- list()

View(set)

for (i in set) {

x <- as.character(a[,i])

x <- x[nchar(x)!=0]

x <- as.character(x)

l[[i]] <-x

}

View(l)

class(l)

save(l,file = "C:/Users/Administrator/Desktop/gene_set.Rdata")

library(GSVA)

library(limma)

load(file = "C:/Users/Administrator/Desktop/gene_set.Rdata")

fpkm <- read.table("DATA.txt", header=T,sep="\t")

rownames(fpkm) <- fpkm[,1]

rows <- rownames(unique(fpkm['NEGE']))

fpkm <- fpkm[rows,]

rownames(fpkm) <- fpkm[,1]

fpkm <- fpkm[,-1]

fpkm[1:3,1:3]

dat <- as.matrix(fpkm)

ssgsea<- gsva(dat,l, method='ssgsea', kcdf='Gaussian',abs.ranking=TRUE)

library(estimate)

filterCommonGenes(input.f="d:/input1.txt", output.f="d:/output1.gct", id="GeneSymbol")

estimateScore(input.ds = "d:/output1.gct",output.ds="d:/estimate_score.gct")

plotPurity(scores="d:/estimate_score.gct", platform="affymetrix")

scores=read.table("d:/estimate_score.gct",skip = 2,header = T)

rownames(scores)=scores[,1]

scores=t(scores[,3:ncol(scores)])

scores

7. GSEA

library(org.Hs.eg.db)

library(clusterProfiler)

library(enrichplot)

df = read.table("gene_diff.txt",header = T)

# df = read.csv("gene_diff.csv",header = T)

head(df)

dim(df)

df_id<-bitr(df$SYMBOL,

fromType = "SYMBOL",

toType = "ENTREZID",

OrgDb = "org.Hs.eg.db")

df_all<-merge(df,df_id,by="SYMBOL",all=F)

head(df_all)

dim(df_all)

df_all_sort <- df_all[order(df_all$logFC, decreasing = T),]

gene_fc = df_all_sort$logFC

head(gene_fc)

names(gene_fc) <- df_all_sort$ENTREZID

head(gene_fc)

KEGG <- gseKEGG(gene_fc, organism = "hsa")

sortKEGG<-KEGG[order(KEGG$enrichmentScore, decreasing = T),]

head(sortKEGG)

dim(sortKEGG)

write.table(sortKEGG,"gsea_sortKEGG.txt")

paths <- c("hsa05204", "hsa00982", "hsa05150", "hsa00980","hsa00983", "hsa03010", "hsa00190", "hsa05418")

gseaplot2(KEGG,paths, pvalue_table = FALSE)

8. LASSO-COX regression analysis

library(glmnet)

library(survival)

x=as.matrix(exp2[,3:ncol(exp2)])

y=data.matrix(Surv(exp2$time,exp2$status))

fit = glmnet(x,y,family = 'cox',maxit = 1000)

plot(fit,xvar='lambda',label = TRUE)

cvfit = cv.glmnet(x,y,family = 'cox', maxit =1000)

pdf('gmy_cvfit.pdf')

plot(cvfit)

abline(v = log(c(cvfit$lambda.min,cvfit$lambda.1se)),lty = 'dashed')

dev.off()

coef = coef(fit,s=cvfit$lambda.min)

index = which(coef != 0)

actcoef = coef[index]

lassoGene = row.names(coef)[index]

lassoGene=c('rfs','event',lassoGene)

lassosigexp = exp2[,lassoGene]

lassosigexp = cbind(id = row.names(lassosigexp),lassosigexp)

write.table(lassosigexp,file = 'lassosigexp.txt',sep = '\t',row.names = F,quote = F)

9. Independent prognostic factor

library(survival)

bioForest=function(coxFile=null, forestFile=null, forestCol=null){

rt <- read.table(coxFile, header=T, sep="\t", check.names=F, row.names=1)

gene <- rownames(rt)

hr <- sprintf("%.3f",rt$"HR")

hrLow <- sprintf("%.3f",rt$"HR.95L")

hrHigh <- sprintf("%.3f",rt$"HR.95H")

Hazard.ratio <- paste0(hr,"(",hrLow,"-",hrHigh,")")

pVal <- ifelse(rt$pvalue<0.001, "<0.001", sprintf("%.3f", rt$pvalue))

pdf(file=forestFile, width=6.5, height=4.5)

n <- nrow(rt)

nRow <- n+1

ylim <- c(1,nRow)

layout(matrix(c(1,2),nc=2),width=c(3,2.5))

xlim = c(0,3)

par(mar=c(4,2.5,2,1))

plot(1,xlim=xlim,ylim=ylim,type="n",axes=F,xlab="",ylab="")

text.cex=0.8

text(0,n:1,gene,adj=0,cex=text.cex)

text(1.5-0.5*0.2,n:1,pVal,adj=1,cex=text.cex);text(1.5-0.5*0.2,n+1,'pvalue',cex=text.cex,font=2,adj=1)

text(3.1,n:1,Hazard.ratio,adj=1,cex=text.cex);text(3.1,n+1,'Hazard ratio',cex=text.cex,font=2,adj=1)

par(mar=c(4,1,2,1),mgp=c(2,0.5,0))

xlim = c(0,max(as.numeric(hrLow),as.numeric(hrHigh)))

plot(1,xlim=xlim,ylim=ylim,type="n",axes=F,ylab="",xaxs="i",xlab="Hazard ratio")

arrows(as.numeric(hrLow),n:1,as.numeric(hrHigh),n:1,angle=90,code=3,length=0.05,col="darkblue",lwd=3)

abline(v=1, col="black", lty=2, lwd=2)

boxcolor = ifelse(as.numeric(hr) > 1, forestCol, forestCol)

points(as.numeric(hr), n:1, pch = 15, col = boxcolor, cex=2)

axis(1)

dev.off()

}

indep=function(expFile=null,cliFile=null,uniOutFile=null,multiOutFile=null,uniForest=null,multiForest=null){

exp=read.table(expFile, header=T, sep="\t", check.names=F, row.names=1) #读取表达文件

cli=read.table(cliFile, header=T, sep="\t", check.names=F, row.names=1) #读取临床文件

sameSample=intersect(row.names(cli),row.names(exp))

exp=exp[sameSample,]

cli=cli[sameSample,]

rt=cbind(exp, cli)

uniTab=data.frame()

for(i in colnames(rt[,3:ncol(rt)])){

cox <- coxph(Surv(futime, fustat) ~ rt[,i], data = rt)

coxSummary = summary(cox)

uniTab=rbind(uniTab,

cbind(id=i,

HR=coxSummary$conf.int[,"exp(coef)"],

HR.95L=coxSummary$conf.int[,"lower .95"],

HR.95H=coxSummary$conf.int[,"upper .95"],

pvalue=coxSummary$coefficients[,"Pr(>|z|)"])

)

}

write.table(uniTab,file=uniOutFile,sep="\t",row.names=F,quote=F)

bioForest(coxFile=uniOutFile, forestFile=uniForest, forestCol="green")

uniTab=uniTab[as.numeric(uniTab[,"pvalue"])<1,]

rt1=rt[,c("futime", "fustat", as.vector(uniTab[,"id"]))]

multiCox=coxph(Surv(futime, fustat) ~ ., data = rt1)

multiCoxSum=summary(multiCox)

multiTab=data.frame()

multiTab=cbind(

HR=multiCoxSum$conf.int[,"exp(coef)"],

HR.95L=multiCoxSum$conf.int[,"lower .95"],

HR.95H=multiCoxSum$conf.int[,"upper .95"],

pvalue=multiCoxSum$coefficients[,"Pr(>|z|)"])

multiTab=cbind(id=row.names(multiTab),multiTab)

write.table(multiTab,file=multiOutFile,sep="\t",row.names=F,quote=F)

bioForest(coxFile=multiOutFile, forestFile=multiForest, forestCol="red")

}

indep(expFile="expTime.txt",

cliFile="clinical.txt",

uniOutFile="uniCox.txt",

multiOutFile="multiCox.txt",

uniForest="uniForest.pdf",

multiForest="multiForest.pdf")

10. Nomogram construction

library(survival)

library(survminer)

library(rms)

data=read.table("liexian.txt",header = T,sep="\t")

dd <- datadist(data)

options(datadist="dd")

f <- cph(Surv(Time, Status) ~ T+Cancer_Status+RISK, x=T, y=T, surv=T, data=data,time.inc=1)

surv <- Survival(f)

nom <- nomogram(f, fun=list(function(x) surv(1, x), function(x) surv(3, x), function(x) surv(5, x)),

lp=F, funlabel=c("1-year survival", "3-year survival", "5-year survival"), maxscale=100,

fun.at=c(0.95, 0.9, 0.8, 0.7, 0.6, 0.5))

plot(nom,xfrac=.5,cex.axis=1,cex.var=1)

validate(f, method="boot", B=1000, dxy=T)

rcorrcens(Surv(Time, Status) ~ predict(f), data = data)

library(boot)

c_index <- function(data,indices){

dat <- data[indices,]

vames<- c("chemoresponse","Risk")

FML <- as.formula(paste('Surv(Time, Status)~',paste(vames, collapse = "+")))

fit<- coxph(FML,data =dat )

pr1<-predict(fit,newdata=dat)

Cindex=rcorrcens(Surv(time, status) ~ pr1, data =dat)[1]

Cindex=1-Cindex

Cindex

}

c_index(data,1:100)

results <- boot(data=data, statistic=c_index, R=500)

boot.ci(results,conf = 0.95)

par(mfrow = c(1,3))

f1 <- cph(Surv(Time, Status) ~ T+Cancer_Status+RISK, x=T, y=T, surv=T, data=data, time.inc=1)

cal1 <- calibrate(f1, cmethod="KM", method="boot", u=1,m=43,B=1000)

par(mar=c(6,5,1,2),cex = 1.0)

plot(cal1,lwd=2,lty=1,subtitles = F,

errbar.col=c(rgb(0,0,0,maxColorValue=255)),

xlim=c(0,1),ylim=c(0,1),

xlab="Predicted 1-year Overall Survival",ylab="Actual 1-year Overall Survival",

col=c(rgb(255,0,0,maxColorValue=255))

)

abline(0,1,lty=3,lwd=2,col="blue")

f3 <- cph(Surv(Time, Status) ~ T+Cancer_Status+RISK, x=T, y=T, surv=T, data=data, time.inc=3)

cal3 <- calibrate(f3, cmethod="KM", method="boot", u=3,m=43,B=1000)

par(mar=c(6,5,1,2),cex = 1.0)

plot(cal3,lwd=2,lty=1,subtitles = F,

errbar.col=c(rgb(0,0,0,maxColorValue=255)),

xlim=c(0,1),ylim=c(0,1),

xlab="Predicted 3-year Overall Survival",ylab="Actual 3-year Overall Survival",

col=c(rgb(255,0,0,maxColorValue=255))

)

abline(0,1,lty=3,lwd=2,col="blue")

f5 <- cph(Surv(Time, Status) ~ T+Cancer_Status+RISK, x=T, y=T, surv=T, data=data, time.inc=5)

cal5 <- calibrate(f5, cmethod="KM", method="boot", u=5,m=43,B=1000)

par(mar=c(6,5,1,2),cex = 1.0)

plot(cal5,lwd=2,lty=1,subtitles = F,

errbar.col=c(rgb(0,0,0,maxColorValue=255)),

xlim=c(0,1),ylim=c(0,1),

xlab="Predicted 5-year Overall Survival",ylab="Actual 5-year Overall Survival",

col=c(rgb(255,0,0,maxColorValue=255))

)

abline(0,1,lty=3,lwd=2,col="blue")

11. Mutation analysis

library("maftools")

annovarToMaf("All.anno.hg19_multianno.txt", Center = NULL, refBuild = "hg19", tsbCol = 'Tumor_Sample_Barcode', table = "refGene", ens2hugo = FALSE, basename = "All", sep = "\t", MAFobj = FALSE, sampleAnno = NULL)

pdf("oncoplot.pdf", width=6, height=6)

oncoplot(maf =maf, fontSize = 0.45 ,showTumorSampleBarcodes = T,SampleNamefontSize=0.7,titleFontSize=1.2,legendFontSize=0.7,removeNonMutated=F,writeMatrix=T)

dev.off()

12. Drug sensitivity analysis

library(pRRophetic)

library(ggplot2)

library(cowplot)

dat <- read.table("EXP.txt",sep = "\t",row.names = 1,header = T,stringsAsFactors = F,check.names = F)

ann <- read.table("group.txt",sep = "\t",row.names = 1,header = T,stringsAsFactors = F,check.names = F)

table(ann$group)

GCP.drug <- read.table("drug.txt",header =F,stringsAsFactors = F)

GCP.drug <- GCP.drug$V1

jco <- c("#BC3C29", "#0072B5")

GCPinfo <- GCP.IC50 <- GCP.expr <- cvOut <- predictedPtype <- predictedBoxdat <- list()

plotp <- list()

for (drug in GCP.drug) {

set.seed(1248103)

cat(drug," starts!\n")

predictedPtype[[drug]] <- pRRopheticPredict(testMatrix = as.matrix(dat[,rownames(ann)]),

drug = drug,

tissueType = "allSolidTumors",

selection = 1)

if(!all(names(predictedPtype[[drug]])==rownames(ann))) {stop("Name mismatched!\n")}

predictedBoxdat[[drug]] <- data.frame("est.ic50"=predictedPtype[[drug]],

"group"=ann$group,

row.names = names(predictedPtype[[drug]]))

predictedBoxdat[[drug]]$group <- factor(predictedBoxdat[[drug]]$group,levels = c("H","L"))

p <- ggplot(data = predictedBoxdat[[drug]], aes(x=group, y=est.ic50))

p <- p + geom_boxplot(aes(fill = group)) +

scale_fill_manual(values = jco[1:length(unique(ann$group))]) +

theme(legend.position="none") +

theme(axis.text.x = element_text(angle = 45, hjust = 1,size = 12),plot.title = element_text(size = 12, hjust = 0.5)) +

xlab("") + ylab("Estimated IC50") +

ggtitle(drug)

plotp[[drug]] <- p

cat(drug," has been finished!\n")

write.csv(predictedPtype[[drug]],file=paste(drug,"est.ic50.csv",sep="_"))

ggsave(paste(drug,"boxplot of predicted IC50.pdf",sep="_"), width = 4, height = 4)

}

p <- vector()

for (drug in GCP.drug) {

tmp <- wilcox.test(as.numeric(predictedBoxdat[[drug]][which(predictedBoxdat[[drug]]$group %in% "H"),"est.ic50"]),

as.numeric(predictedBoxdat[[drug]][which(predictedBoxdat[[drug]]$group %in% "L"),"est.ic50"]),alternative = "less")$p.value

p <- append(p,tmp)

}

names(p) <- GCP.drug

write.table(p,"output_pvalue.txt", quote = F, sep = "\t")
