# Supplementary figures and images for: A scoring model based on bacterial lipopolysaccharide-related genes to predict prognosis in NSCLC
Source: Front Genet. 2024 Nov 14;15:1408000. doi: 10.3389/fgene.2024.1408000 (PMC11602480; doi:10.3389/fgene.2024.1408000)

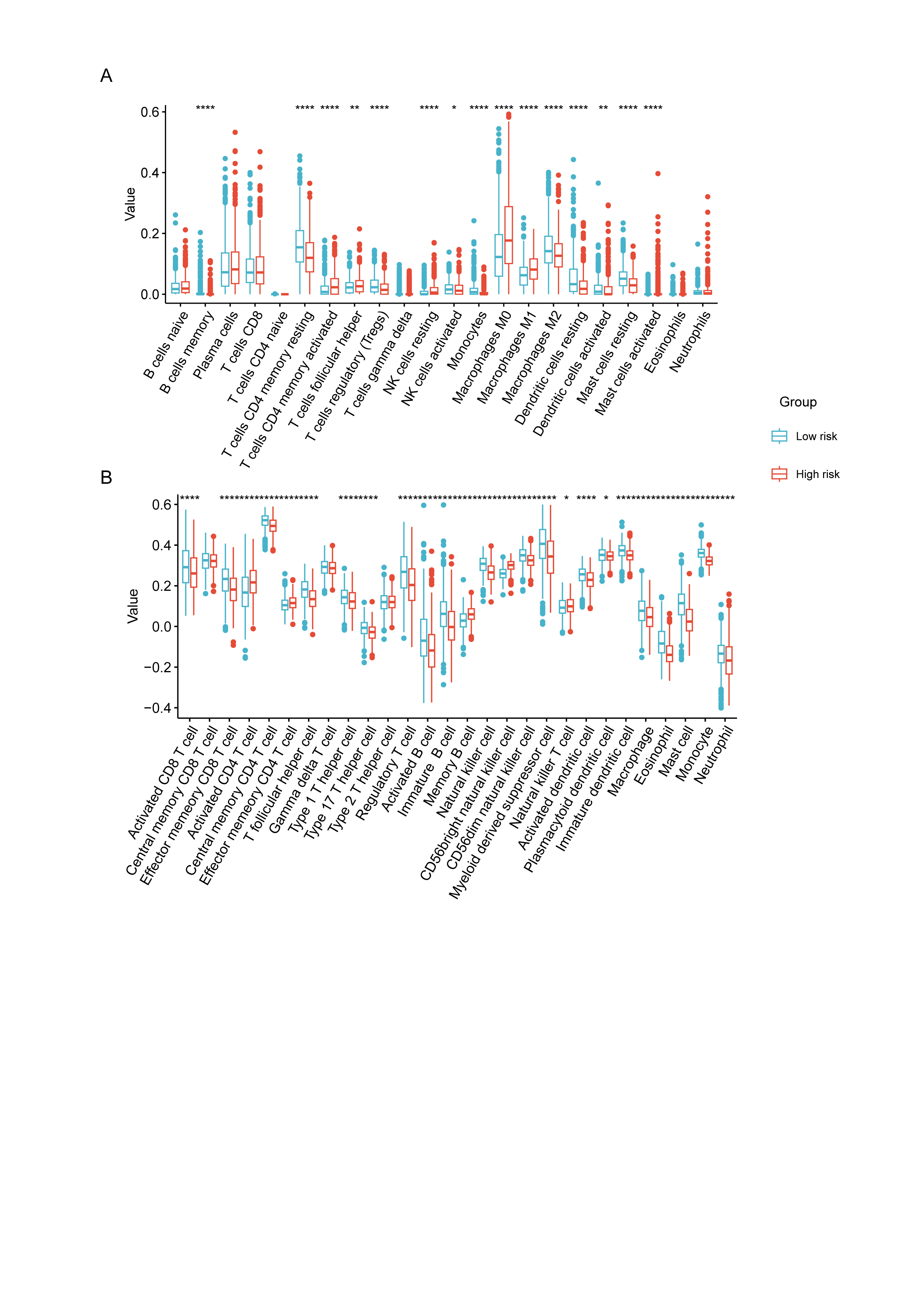

Supplement: Supplementary file 4 [file Image2.tif]

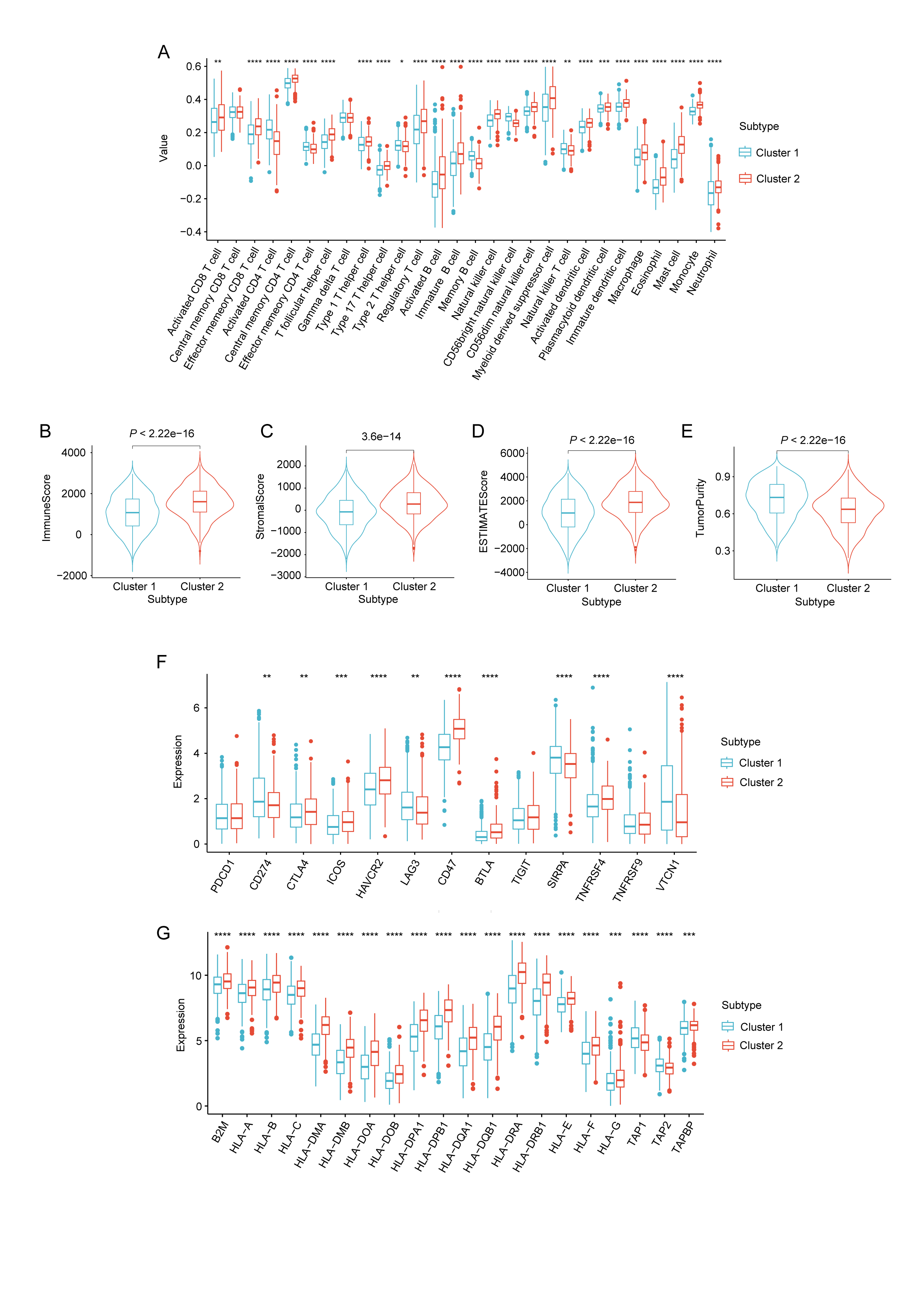

Supplement: Supplementary file 5 [file Image1.tif]
